# Management of Latent Tuberculosis Infections in Australia and New Zealand: A Review of Current Practice

**DOI:** 10.1155/2010/284028

**Published:** 2010-12-29

**Authors:** Justin T. Denholm, Emma S. McBryde

**Affiliations:** ^1^Victorian Infectious Diseases Service, Royal Melbourne Hospital, Grattan Street, Parkville, VIC, 3050, Australia; ^2^Department of Medicine, University of Melbourne, Parkville, VIC, 3010, Australia; ^3^Anton Breinl Centre, James Cook University, Townsville, QLd, 4810, Australia

## Abstract

*Aim*: To survey practices in the diagnosis and management of latent tuberculosis infection (LTBI) in Australia and New Zealand. *Methods*: Infectious diseases and respiratory physicians and trainees were invited to complete an online questionnaire concerning various aspects of LTBI management. *Results*: The questionnaire was completed by 126 clinicians self-reporting regular management of LTBI. Respondents were experienced physicians, with 95/126 (75.4%) having managed LTBI for more than 5 years. Forty-seven (37.3%) reported seeing more than 5 patients per month for assessment of LTBI. Substantial variation among clinicians was reported in relation to a number of common clinical scenarios. For instance, while 52/126 (43.7%) informed patients that the incidence of severe hepatotoxicity related to isoniazid monotherapy was 0.1–0.5%, 21/126 (15.7%) thought it was *>*5%. 36/126 (28.6%) clinicians would proceed with TNF-***α***therapy following an indeterminate screening: interferon-***γ***assay, while 78/126 (61.9%) would perform further investigations and 12/126 (9.5%) would initiate isoniazid therapy. Follow-up intervals during therapy varied from 1–3 monthly, with liver function testing performed routinely by 89/126 (70.6%). *Conclusion*: This study demonstrated a large degree of variation in clinical practice of LTBI management in Australia and New Zealand. Strategies for increasing uniformity of practice are required, including improved guidelines and physician education.

## 1. Introduction

*Mycobacterium tuberculosis* (TB) infection is a significant cause of morbidity and mortality worldwide, with an estimated 2 billion people at risk for reactivation [1]. TB can remain dormant for decades before reactivation, although most latent infections in nonimmunosuppressed adults will never reactivate [2]. The risk of reactivation in patients diagnosed with LTBI can be substantially reduced through appropriate use of antituberculosis medications; however, treatment is lengthy and involves some risk of serious side effects, particularly hepatotoxicity [3, 4]. 

In Australia and New Zealand, the largest burden of TB infection occurs due to LTBI reactivation in immigrants from high-prevalence countries. No routine screening for LTBI is mandated at the time of immigration, although this is recommended for recently arrived refugees [5]. Treatment of LTBI is largely performed by infectious diseases and respiratory physicians, in part due to restrictions on prescribing antituberculosis medications by nonspecialists. These clinicians practices and their uniformity are of interest, as effective identification and treatment of LTBI is likely to result in decreased incidence of active TB infection, particularly in these low-transmission settings. 

Although increasing evidence to guide various aspects of LTBI is available, there remain many areas of management where clinical trial data are lacking. Optimal duration of therapy, for instance, remains controversial, with conflict among international guidelines. Similar uncertainty exists in regards to appropriate monitoring while on therapy, the use of tuberculin skin testing or interferon-gamma release assays (IGRA), and a raft of other clinical questions. Given the paucity of data on many LTBI treatment issues, it is likely that approaches to management vary among treating clinicians. However, no broad survey of management practices has previously been undertaken in Australia and New Zealand, and the extent to which a standard approach exists is not known. This study undertook to survey current practices of physicians in Australia and New Zealand engaged in the management of LTBI and consider their uniformity and potential impact on public health aspects of tuberculosis.

## 2. Methods

All currently practicing infectious diseases and respiratory physicians and trainees were eligible to participate in this study. Potential participants were contacted through existing Email distribution lists for the relevant professional organisations (Australasian Society of Infectious Diseases and the Thoracic Society of Australia and New Zealand). Potential participants were invited to complete an internet-based questionnaire if their usual clinical practice involved management of adult patients with known or suspected latent TB infection.

Respondents to the initial E-mail contact were directed to an independent online survey site. Participants information was collected concerning type of training (infectious diseases or respiratory physicians) and extent of involvement with the management of latent tuberculosis infection (number of patients seen and years of practice). Participants were then asked to respond to 12 short scenarios related to latent TB infection and management. All responses were of a “best-answer” type to four multiple-choice options. 

Survey responses were recorded, and basic descriptive statistics were performed using Excel. Responses were then combined into binary options for intragroup comparison, and two-tailed *P* values were calculated using *χ* ^2^ testing or Fisher's exact test for values <5. 

This study was approved by the Melbourne Health Human Research Ethics Committee as a quality assurance activity. No individual identifying information was recorded or collected, and participation was voluntary.

## 3. Results

### 3.1. Participants

A total of 126 respiratory and infectious diseases clinicians completed the questionnaire. 95 participants were physicians, representing 9.6% of all infectious diseases and respiratory physicians in Australia and New Zealand (386 infectious diseases physicians and 599 respiratory physicians; data supplied by relevant specialist societies). Participants information is shown in Table 1.

Respondents were generally experienced clinicians, with 95/126 (75.4%) being consultant physicians and 70/126 (55.6%) reporting having managed LTBI for more than 5 years. Currently, most clinicians were in relatively low-volume practice, with 79/126 (62.7%) seeing less than 5 patients for assessment of LTBI per month. 

Responses to scenarios have been grouped below by theme.

### 3.2. Diagnosis

Overall, clinicians were more likely to use an IGRA than TST for the diagnosis of LTBI. Several questions offered a choice between the two tests in a variety of situations, with respondents opting for IGRA more frequently (62.8% versus 37.2%; *P* < .05). Most clinicians would use an IGRA for diagnosis of LTBI in patients known to have had BCG vaccination, and while clinicians frequently reported using an IGRA after a positive TST (84/126, 66.7%), no respondent performed TST following a positive IGRA. Following an indeterminate IGRA, clinicians were more likely to perform TST (32.9%) than to repeat IGRA (24.2%). Additionally, a small number of respondents (4/126, 3.2%) indicated that they would use IGRA becoming negative as a guide to successful therapy.

When presented with a 25-year-old patient with a positive TST and a history of childhood BCG vaccination, 84/126 (66.7%) of clinicians reported that they would proceed to an interferon-gamma assay before considering whether treatment for LTBI was appropriate. 24 (19.0%) would review in 3 months, while 15 (11.9%) would start isoniazid therapy immediately.

An immigrant from a high-prevalence country being screened for LTBI prior to starting infliximab was found to have an indeterminate interferon-gamma response. 28.6% (36/126) of clinicians reported that they would proceed with infliximab therapy and observe, while 9.5% (12/126) would initiate LTBI therapy prior to infliximab. The remainder indicated they would perform further tests to diagnose LTBI, either repeating IGRA (17/78; 21.8%) or TST (61/78; 78.2%).

In a separate question, respondents were also asked how they would manage an indeterminate interferon-gamma test in a 40-year-old recent immigrant from a country with high TB prevalence. 66/126 (52.4%) said they would perform another test (44/126 would repeat IGRA, 22/126 would perform TST), while 47/126 (37.3%) would review clinically and 13/126 (10.3%) would initiate treatment for LTBI.

### 3.3. Initiating LTBI Therapy

Respondents were asked to choose appropriate therapy for confirmed LTBI in a patient exposed to known isoniazid-monoresistant TB. 77/126 (61.1%) prescribed rifampicin (600 mg daily for 4/12), while 20/126 (15.9%) opted for an isoniazid-based regimen (300 mg for 6 or 9/12). 23% (29/126) said they would prescribe combination therapy with ethambutol and pyrazinamide for 4/12.

A 14-week pregnant patient with recent exposure to a case of active tuberculosis was found to have a positive interferon-gamma assay. 41/126 (32.5%) indicated that they would delay management until after delivery, while the remainder suggested some earlier investigation or therapy. Of those who would proceed with management in pregnancy, 66/85 would perform a CXR to exclude active TB, while the remaining 19/85 would immediately start treatment for LTBI.

### 3.4. Side Effects of LTBI Therapy

Respondents were asked to estimate the likelihood of a young man developing severe hepatotoxicity (Alanine transaminase (ALT) >5 times ULN) while receiving isoniazid monotherapy. Responses differed considerably: while 52/126 (43.7%) believed the incidence was 0.1–0.5%, 21/126 (15.7%) thought it was >5%. A number of large studies and reviews of isoniazid monotherapy have concluded that the incidence of serious hepatitis (ALT >5 ULN) is between 0.1 and 0.56%; a figure taken as the correct answer to this question [6–8]. Clinicians who reported seeing more than 5 patients/month for the management of LTBI were very likely to answer correctly (32/37, 86.5%). They were significantly more likely to respond correctly than clinicians who had more general experience (>5 years practice) but were assessing less than 5 patients/month for LTBI (20/51, 39.2%; *P* < .0001) or the overall group (*P* < .0001).

When managing an asymptomatic patient with an ALT that became elevated while receiving isoniazid, but remained less than 3 times the upper limit of normal, 84/126 (66.7%) elected to continue therapy without modification. Interestingly, 13/126 respondents reported that they would dose-reduce isoniazid in this setting. This strategy was more common in respiratory practitioners (11/60, 18%) than in infectious diseases practitioners (2/66, 3%; *P* < .001) and was particularly common amongst respiratory trainees (5/10; 50%).

### 3.5. Review and Completing Therapy

**Figure 1:**
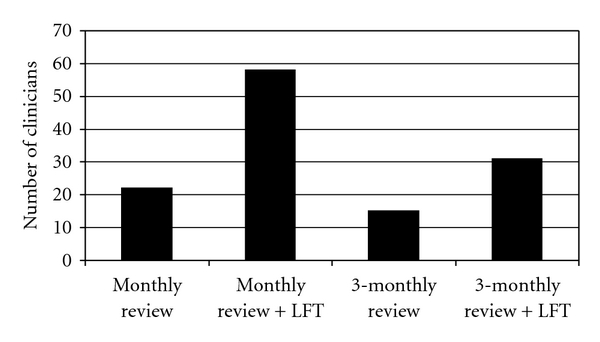
Followup during Treatment.

Clinicians were asked how frequently they would routinely review asymptomatic young patients with normal baseline liver function testing (LFT) and whether they would routinely perform serial LFT during therapy. Results are shown in Figure 1. No significant differences were seen between infectious diseases and respiratory physicians.

Respondents were also asked to consider a 45-year-old woman who was found to have missed 50 doses from a planned 9/12 course of isoniazid therapy. 55/126 (43.7%) of clinicians would cease therapy as planned, with the remainder extending the course. 43/126 reported that they would continue for a further 3/12, while 24/126 would continue until all planned doses had been administered.

## 4. Discussion

This study found that participating clinicians in Australia and New Zealand reported significant variation in a variety of common practices related to the diagnosis and management of LTBI. These variations included testing algorithms, treatment selection and duration, and follow-up strategies, as well as estimated frequency of adverse effects on therapy. Such variation is likely to detract from the public health impact resulting from LTBI management, and appropriate strategies to improve practice should be considered.

To our knowledge, this is the first reported audit of broad clinical management of LTBI, although smaller audits have reviewed specific aspects such as screening pre-TNF*α* inhibitor use [9]. Participants included both respiratory and infectious diseases clinicians, were recruited across all Australian states and territories and New Zealand, and provided actual practice information about current clinical management of LTBI in Australia and New Zealand. Due to the “blanket” distribution of the survey to members of the professional societies of infectious diseases and respiratory physicians, our study is limited by an inability to characterise nonresponders and participant self-reporting. However, our survey captured approximately 10% of all infectious diseases and respiratory physicians in Australia and New Zealand, which is anecdotally consistent with the proportion conducting LTBI management, and potential subjects were asked to self-select on the basis of having clinical practices including regular management of LTBI. We believe that it is likely that the group sampled in this study provides a more helpful assessment of actual LTBI management in Australia and New Zealand than a more complete survey of infectious diseases and respiratory clinicians would have done.

The variation in practice seen in this study is likely to reflect several issues. Although some aspects of LTBI management are well studied, such as treatment-limiting side effects of chemotherapy, a paucity of data exists on certain issues, such as best practice follow-up strategies. Some discrepancies in practice may reflect a lack of awareness, particularly amongst less experienced clinicians. Additionally, approaches to LTBI management have changed considerably over several decades, and clinicians not actively involved in regular clinical practice may not be aware of recent changes.

Optimal management of LTBI has the potential to result in a variety of public health benefits. It should minimise the risk of reactivation TB infection, decreasing both the health burden of infection and the degree of secondary transmission. It may also assist in preventing the emergence of multidrug resistant strains of TB and be applied in ways that are both acceptable and cost effective. In order to accomplish these aims, however, strategies must be rational and consistently applied. Several aspects of this study highlight current practice issues that have the potential to work against these goals and require further comment.

A number of clinicians in this survey reported dose reduction of isoniazid when managing isoniazid-associated hepatotoxicity. Dose reduction of isoniazid has been demonstrated to decrease early bactericidal activity when doses less than 300 mg/day are used, and such regimens are not recommended [10, 11]. A minority of clinicians also reported using isoniazid-based regimens for the treatment of isoniazid-resistant LTBI infection, a practice that has been shown to be ineffective [12]. Effective public health strategies for addressing LTBI require consistent and rational prescribing practices. Educational strategies related to rational therapeutic prescribing may be of value in strengthening such programs, particularly targeting less experienced prescribers.

Rational assessment of the cost effectiveness of any LTBI strategy is hampered by the lack of a gold-standard diagnostic test [13, 14]. Such difficulties may be further compounded by several aspects of the responses observed in this present study. For instance, significant variation was reported in the frequency of follow-up review and tests of liver function and damage (LFT) in patients on isoniazid. Little evidence exists to strongly support specific practices, which is reflected in variation among international guidelines. For instance, the American Thoracic Society recommends monthly clinical review without repeated LFT in low-risk patients, while British guidelines do not offer a specific review program [15, 16]. Different review programs are associated with substantial variation in cost, and New Zealand LTBI guidelines recommend clinical review and LFT be performed 3 times monthly, based specifically on cost effectiveness data [17]. Cost-effectiveness issues also arise through the rational use of new diagnostic technologies such as IGRA. A small number of respondents reported using IGRA to determine whether LTBI therapy had been successful, an application for which evidence to date is not supportive of [18]. A number of clinicians also reported performing TST following indeterminate IGRA, which would be expected to have a low yield based on published comparisons and unlikely to be cost effective [19]. Finally, there is some evidence that performing multiple diagnostic tests may interfere with individual test performance, a finding which would further impair rational approaches to ensuring cost-effective LTBI therapy [20].

## 5. Conclusions

The majority of cases of active tuberculosis infection in Australia and New Zealand are reactivation from latent disease, most commonly acquired in high-prevalence countries. Effective strategies for managing LTBI can reasonably be expected to reduce the burden of subsequent active infections. Such strategies must, however, be rational and consistently applied. Inconsistency in practice related to the management of latent TB infection is likely to initially affect immigrants and refugees disproportionately given that they bear the burden of TB infection in Australia and New Zealand today. Difficulties involved in managing LTBI in these populations may include cultural and linguistic barriers, coexisting medical conditions, and geographic relocation and should not be further compounded by unnecessary practice variation.

Recognition of significant differences surrounding many areas of current management of LTBI in Australia and New Zealand is important and should provide impetus for developing strategies to improve consistency. This survey has shown that current practice is highly variable in key areas, and the development, implementation, and regular updating of guidelines specifically for the Australian and New Zealander context are important. Such guidelines must be developed with input from a variety of stakeholders to assist with ensuring broad acceptability but are likely to be most beneficial if approached uniformly across the region. The results of this study suggest that further education may be useful, particularly for trainees and less experienced clinicians. Finally, further research into the areas of practice for which little data exist is critical and may ultimately be necessary to resolve differences in practice.

## Figures and Tables

**Table 1:** Participants information.

	ID physician (*n* = 45)	Respiratory physician (*n* = 50)	ID trainee (*n* = 21)	Respiratory trainee (*n* = 10)
Duration of practice (years)				

<2	0	1	11	7
2 to 5	13	10	10	3
6 to 10	14	17	0	0
>10	18	22	0	0

Number of patients/month				

<2	22	28	7	3
2 to 5	11	7	7	4
6 to 10	6	7	5	2
>10	6	8	2	1
